# enviRule: an end-to-end system for automatic extraction of reaction patterns from environmental contaminant biotransformation pathways

**DOI:** 10.1093/bioinformatics/btad407

**Published:** 2023-06-24

**Authors:** Kunyang Zhang, Kathrin Fenner

**Affiliations:** Department of Environmental Chemistry, Eawag, Dübendorf 8600, Switzerland; Department of Chemistry, University of Zürich, Zürich 8057, Switzerland; Department of Environmental Chemistry, Eawag, Dübendorf 8600, Switzerland; Department of Chemistry, University of Zürich, Zürich 8057, Switzerland

## Abstract

**Motivation:**

Transformation products (TPs) of man-made chemicals, formed through microbially mediated transformation in the environment, can have serious adverse environmental effects, yet the analytical identification of TPs is challenging. Rule-based prediction tools are successful in predicting TPs, especially in environmental chemistry applications that typically have to rely on small datasets, by imparting the existing knowledge on enzyme-mediated biotransformation reactions. However, the rules extracted from biotransformation reaction databases usually face the issue of being over/under-generalized and are not flexible to be updated with new reactions.

**Results:**

We developed an automatic rule extraction tool called enviRule. It clusters biotransformation reactions into different groups based on the similarities of reaction fingerprints, and then automatically extracts and generalizes rules for each reaction group in SMARTS format. It optimizes the genericity of automatic rules against the downstream TP prediction task. Models trained with automatic rules outperformed the models trained with manually curated rules by 30% in the area under curve (AUC) scores. Moreover, automatic rules can be easily updated with new reactions, highlighting enviRule’s strengths for both automatic extraction of optimized reactions rules and automated updating thereof.

**Availability and implementation:**

enviRule code is freely available at https://github.com/zhangky12/enviRule.

## 1 Introduction

Environmental biotransformation of a variety of organic contaminants plays an important role in chemical risk management (Kern *et al.* 2010, Olvera-Vargas *et al.* 2016), bioremediation of contaminated sites (de Lorenzo *et al.* 2008), and the development of green chemical alternatives (Moermond *et al.* 2022). In most cases, the bioactivity of transformation products (TPs) formed as part of the biotransformation process is mitigated through the structural changes introduced. However, examples have been reported where TPs exhibit equal or even higher bioactivities due to the conservation or formation of toxicophore structures in TPs, resulting in greater ecological risks compared to the parent compounds (Cwiertny *et al.* 2014). For example, decreases in the diversity and function of soil microorganisms were observed in a soil treated with 3,5-dichloroaniline (3,5-DCA), but not in the soil treated with its parent pesticide iprodione, suggesting greater toxicity of 3,5-DCA to soil microorganisms (Vasileiadis *et al.* 2018). Hence TPs should be included into hazard and risk assessment to obtain a comprehensive metric for the biological effects of contaminants. Yet, the identification and structural characterization of TPs is challenging, especially in environmental samples (Hubert *et al.* 2017). Typically, analysis by liquid chromatography-high resolution mass spectrometry (LC-HRMS) suspect or nontarget screening is required for the identification of formed TPs (Helbling *et al.* 2010, Funke *et al.* 2016), which is time-consuming and labor-intensive. Therefore, *in silico* models that can accurately predict possible biotransformation TPs are an essential tool for the comprehensive ecological risk assessment of environmental contaminants.

Substantial efforts have been made to enable the *in silico* prediction of TPs from contaminant biotransformation by environmental microbial communities, and a number of rule-based systems have been developed, including enviPath (Wicker *et al.* 2016), the chemical transformation simulator by the US EPA (https://scholarsarchive.byu.edu/iemssconference/2016/Stream-A/19/), and Biotransformer (Djoumbou-Feunang *et al.* 2019, Wishart *et al.* 2022). All of these *in silico* prediction tools combine both domain knowledge and machine learning models to predict and prune biotransformation pathways. Although rule-free models (i.e. sequence-based models) have also been shown to achieve considerable success in predicting the outcomes of chemical reactions (Schwaller *et al.* 2019, 2021a, b), and sometimes even outperform rule-based models, they require large amounts of data for their training. This mostly prevents them from being applied on environmental biotransformation reactions, which only have limited sizes of datasets, especially when the reactions are confined to specific environmental matrices, such as soil, water, sediments, and sludge (Satoh *et al.* 2023). Rules significantly facilitate the training of models on small datasets by imparting the existing knowledge on enzyme-mediated biotransformation reactions to models, making rule-based models the currently most suitable approach for predicting TPs formed through environmental biotransformation reactions.

The key challenge in developing rule-based models is the extraction of reaction rules from reaction databases. An exhaustive comparison of rules extracted from enzymatic reactions can be found in a previous report (Ni *et al.* 2021). Among the rules that are currently available online, the majority of them were manually curated, e.g. EAWAG-PPS rules (Gao *et al.* 2010), BNICE rules (Li *et al.* 2004), and MINE rules (Jeffryes *et al.* 2015). Manually curated rules are designed and examined by experts, and the rules can normally explain the most common reactions. However, recent improvements in techniques for elucidating biotransformation reactions at trace contaminant levels, e.g. the increased accessibility of LC-HRMS instruments and data analysis methods, enable biotransformation reactions to be discovered at increasing speed (Zimmermann *et al.* 2019), causing a surge in the volume of datasets. In addition, efforts have been made over the past five years to encode pathway information from publicly available regulatory dossiers. In enviPath specifically, the addition of the EAWAG-SOIL package, containing information on pesticide degradation pathways in soil, to the legacy EAWAG-BBD package has almost doubled the number of available reactions (Latino *et al.* 2017). With the addition of new pathway information, manually curated rules can be limited in representing the newly added reactions for two main reasons. First, due to the expansion of the chemical space covered, new reaction patterns might be present in the new datasets that are not included in existing rules at all. In the other case, similar reaction patterns are already included but not generalized enough to account for enzyme promiscuity on substrates in new reactions. At this point, with manually curated rules, it is very challenging to (i) sort new reactions into one of these two categories, and (ii) find the optimal level of generalization, also called genericity, for each rule.

Therefore, interest has shifted from manually curated rules to automatically extracted rules. Indeed, systems like BNICE and ATLASx, which once used manual rules, have been updated to automatic rules in recent years (Ni *et al.* 2021, MohammadiPeyhani *et al.* 2022). Tools for automatic extraction of rules, based on atom–atom mapping (AAM) for the recognition of reaction centers, have been successfully developed (Duigou *et al.* 2019, Ding *et al.* 2020, Ni *et al.* 2021). Relative to manual rule curation, automatic extraction of rules from reactions can be very fast and the resulting set of rules normally covers reaction patterns more comprehensively. For instance, 4996 automatically extracted rules cover 20 942 biological reactions in Rhea (Ding *et al.* 2020). In addition, when new reactions are added and if it is found that they cannot be covered by any of the existing rules, automatic rule extraction should allow focusing on only those rules that need to be adjusted or created. This can potentially streamline the process of updating existing rules, relative to running extractions again for the combined set of reactions and producing a completely new set of rules. However, this possibility has not yet been extensively explored.

Despite these obvious advantages of automatic rule extraction, the algorithms used are typically fully agnostic of biochemical expert knowledge on enzymes’ substrate specificities. Therefore, the main challenge faced by tools for automatic rule extraction is defining the appropriate genericity of rules (Sveshnikova *et al.* 2022). Although reaction rules can be extracted at different genericity levels by using various extension diameters of reaction centers as demonstrated in RetroRules (Duigou *et al.* 2019), reaction centers are usually not extended (Ni *et al.* 2021) or extended to include only immediately neighboring atoms (Coley *et al.* 2017, Segler and Waller 2017, Ding *et al.* 2020) in order to ensure sufficient coverage. Rules extracted in this way can typically be generalized well to cover unknown reactions as they only specify few neighboring atoms around reaction centers. For example, the rule set with 1224 rules automatically extracted from MetaCyc reactions, which only overlap with 58% of KEGG reactions, can cover 85.2% of all KEGG reactions (Ni *et al.* 2021). However, while their coverage is high, such rules are likely to be falsely triggered on many substrates, resulting in combinatorial explosion when used in pathway predictions (Ding *et al.* 2020). Developing algorithms to balance between coverage and over-generalization in automatic rule extraction is therefore of utmost importance.

Here, we exploit knowledge on observed contaminant biotransformation pathways to explore the possibility of optimizing rule genericity directly against the prediction task at hand. More specifically, we developed an automatic rule generation tool called enviRule that can automatically extract rules from biotransformation reactions, efficiently update automatic rules as new data is added, and determine the optimum genericity of rules for the task of contaminant pathway prediction using the enviPath pathway prediction system (Wicker *et al.* 2016). To that end, we related rule genericity to the pathway prediction performance of machine learning models and determined the optimum rule genericity as yielding the best model performance.

We tested enviRule on the biotransformation reactions contained in the EAWAG-BBD package in enviPath and compared the prediction performance of models trained with the previously manually curated rules and the newly generated automatic rules at both reaction and pathway levels. The automatic rules were then updated with the more recently added reactions contained in the EAWAG-SOIL package to test the applicability of enviRule to deal with the growing number of new reactions.

## 2 System and methods

### 2.1 Biotransformation reaction dataset

Biotransformation reactions reported in the EAWAG-BBD and EAWAG-SOIL packages in enviPath were used for automatic rule generation and updating of rules, respectively. EAWAG-BBD is a mirror data package of the University of Minnesota Biocatalysis/Biodegradation Database (UM-BBD), which was first developed in 1995 (Ellis *et al.* 1999). Over the past two decades, the dataset has grown from 4 to 219 pathways with 1479 reactions (Ellis *et al.* 2006). Most of the data in EAWAG-BBD derives from studies of pure or enrichment cultures. In contrast, EAWAG-SOIL, a rather new data package, contains pesticide biotransformation pathways in soils extracted from the draft assessment reports (DAR) used in pesticide registration that are made publicly available through the European Food Safety Authority (EFSA) (Latino *et al.* 2017). 317 pathways with 2447 reactions observed under aerobic conditions have been extracted from DARs and documented in EAWAG-SOIL. Different from most studies reported in EAWAG-BBD, biotransformation studies reported in EAWAG-SOIL were carried out using ^14^C-labeling, thus likely providing more complete pathways.

### 2.2 Design of enviRule

**Figure 1.**
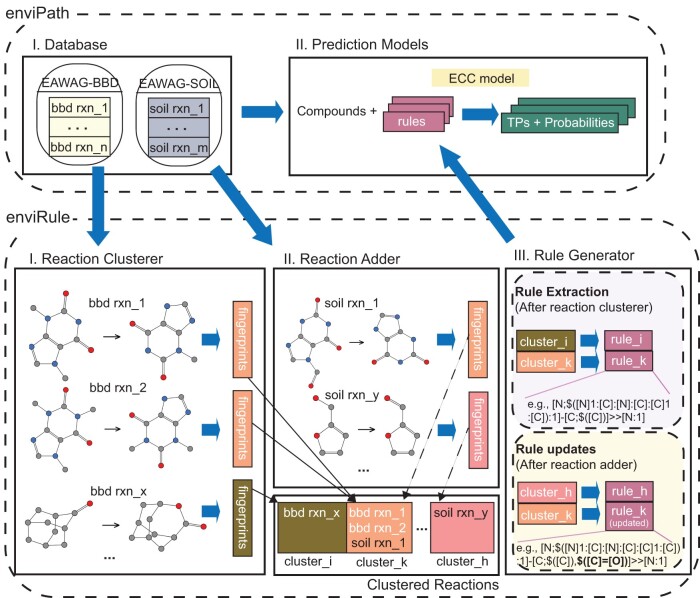
Overview of enviRule. Reactions are first sent to the Reaction Clusterer to calculate reaction fingerprints, which are then used to cluster reactions into different groups. The Rule Generator automatically extract rules from clustered groups. Clustered groups can also be expanded with new reactions that have the same reaction fingerprints, and corresponding rules are updated.

enviRule consists of three modules, namely reaction clusterer, rule generator, and reaction adder, which work closely together to generate and update automatic rules. Reactions are first clustered in reaction clusterer based on reaction centers, then rule generator produces automatic rules for each reaction cluster. When new reactions are added, reaction adder identifies existing rules to be updated and new rules to be created. A schematic overview of enviRule can be found in Fig. 1. SMARTScompareViewer  (Ehmki *et al.*, 2019; Schmidt *et al.*, 2019)  was used to visualize reactions and SMARTS.

#### 2.2.1 Module 1: Reaction clusterer

Reaction clusterer was designed to avoid redundancy in generating automatic rules. Reactions with the same reaction centers are clustered into the same group with the goal of finding one generalized rule for each group in the next step. Reaction clusterer applies the Reaction Decoder Tool (RDT) for calculating AAM and bond changes (Rahman *et al.* 2016). The algorithms have been previously reported in EC-BLAST and are now available in RDT (Rahman *et al.* 2014). Adapted from EC-BLAST, comprehensive reaction fingerprints are created in reaction clusterer to measure reaction similarities. Chemical bonds and atoms in the reaction centers of substrates and products are encoded into multiple fingerprints: a bond formation/cleavage fingerprint, a bond-change fingerprint, which contains all the bonds with changed orders, and a set of reaction-center fingerprints, representing atoms in reaction centers. The reaction centers defined in reaction clusterer include changed atoms and bonds, as well as whole functional groups if any parts of a predefined set of functional groups are involved in reactions. The list of functional groups was adapted from a tool for predicting chemical reaction outcomes with machine learning (Coley *et al.* 2017). Since the Tanimoto coefficient has been widely accepted for calculating the similarity of bit-strings (Holliday *et al.* 2002), it is used as the indicator of reaction similarity in reaction clusterer. Only if the Tanimoto coefficient of reaction fingerprints is 1.0, two reactions are clustered into the same group. The reaction fingerprints of each clustered group are stored for further comparison when new reactions are added through reaction adder. In this project, only reaction groups with at least two reactions were sent to rule generator for automatic rule generation.

#### 2.2.2 Module 2: Rule generator

**Figure 2.**
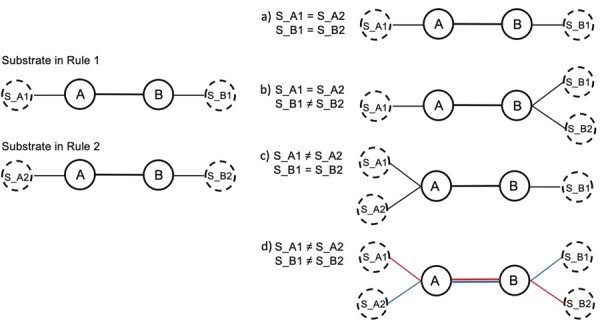
Schematic view of substrate combination. Solid annotated circles are atoms in a reaction center, while dashed annotated circles are substituents. This figure demonstrates the graph combination for a group with two reactions. Four cases are distinguished (a–d) based on whether substituents (S) attached to either atom in the reaction center are equal or not. The left side shows the substrates of two simple rules extracted from the two reactions, and the right side shows three plausible (i.e. a–c) and one incorrect (i.e. d) combined forms of the substrates. Colored lines (i.e. the red and blue lines) in (d) denote unobserved combinations of substituents. In case d, the two reactions cannot be combined into one rule (see also Supplementary Fig. S2 for another concrete example).

In the rule generator, reaction centers are expanded by the breadth first search (BFS) algorithm with adjustable diameters to include neighboring bonds and atoms (i.e. substituents). Rule generator was developed with RDT and CDK. It automatically generates SMIRKS rules (http://www.daylight.com/dayhtml/doc/theory/theory.smirks.html) for each of the reaction groups. In the enviPath prediction system, each rule has a classifier that requires training. To decrease the sparsity of the training data of classifiers, classifiers are built for composite rules. Each composite rule represents one reaction group and may comprise of one or several simple rules extracted from the reactions present in this group. Without a combination strategy, the number of simple rules in a composite rule should equal the number of reactions in the corresponding group. Here, we aimed to minimize the number of simple rules in each composite rule as follows to control redundancy. To minimize the number of rules inside a composite rule we use graph combination to integrate substrates of different reactions without producing unobserved combinations of substituents (i.e. Supplementary Fig. S2), thus avoiding over-generalization of rules. After initializing each substrate as a graph with its original reaction center as backbone and substituents as leaf nodes (Fig. 2), graphs are combined only (i.e. Fig. 2a–c) when no connections are created between two substituents that do not co-exist in any reaction (e.g. substituent S_A1 and substituent S_B2 in Fig. 2d). Graphs are translated into SMARTS after combination.

#### 2.2.2 Module 3: Reaction adder

When new reactions are available, similarly to reaction clusterer, reaction adder clusters them into different groups. In addition, it compares the fingerprints of new reactions with the previously stored fingerprints of reaction groups and adds new reactions into existing reaction groups if the Tanimoto coefficient of their fingerprints is 1.0, otherwise puts them into new groups. Rule generator will be applied on those expanded groups, with existing and new reactions, to produce updated rules that are generalized to cover newly added reactions. For new reactions that cannot be added into any of the existing reaction groups, new rules will be generated by rule generator.

### 2.3 Machine learning models for pathway predictions

Pathway prediction was reduced to a multi-label classification problem in enviPath. The goal is to iteratively predict which rules (i.e. labels) are correctly triggered on a compound and then on its predicted TPs. enviPath used to solve multi-label classification with a binary relevance method (BM) by building independent classifiers for each rule (Wicker *et al.* 2010). Later, BM was replaced by an ensemble chain classifier (ECC) to take the correlation of these classifiers into consideration (Read *et al.* 2011). ECC models are implemented with MEKA (Read *et al.* 2016), which is a multi-label extension of the WEKA machine learning tool (Witten and Frank 2002).

To prepare the training data for ECC models, each compound is represented as a vector consisting of 192 MACCS (Molecular ACCess System) structural fingerprints (Durant *et al.* 2002) and rules that are encoded into a binary string. For training the classifier of a rule, if this rule is triggered on a compound and resulting products are observed in the biotransformation reactions in the dataset, this compound is taken as a positive sample (PS). Conversely, if the rule is triggered but products cannot be observed, this compound is a negative sample (NS).

### 2.4 Performance evaluation

#### 2.4.1 Genericity of automatic rules

The range of compounds a rule can be triggered on depends on how specific this rule is. This characteristic of a rule is also called genericity and is evaluated as follows: where NS represents the number of negative samples used for training the classifier of a rule, while PS stands for the number of positive samples. Genericity, i.e. the ratio of negative to positive samples, significantly affects the training of models and their prediction performance. A specific genericity of a rule can be achieved in rule generator by iteratively adjusting the diameters for the extension of reaction centers or by adding explicit hydrogens. To achieve a genericity target value, a rule is iteratively adjusted and evaluated against all the training compounds to count the number of negative samples and positive samples, until the ratio is as close as possible to the target genericity. For example, if the current genericity of a rule is greater than the target genericity, either extension diameters will be increased or explicit hydrogens will be added by the rule generator to generate a more specific version of this rule, and vice versa.


(1) Genericity=NSPS

The optimum genericity of rules in this project was determined for the task of contaminant pathway prediction using the enviPath pathway prediction system. Specifically, prediction models were trained on 80%of data with 6 sets of automatic rules whose genericities were predefined at different genericity levels of 0, 1, 5, 10, 20, and 50. The genericity corresponding to the model yielding the highest predicted probabilities for the experimentally observed products in the remaining 20% test reactions is selected as optimum genericity. Once the optimum genericity is determined, extension diameters and explicit hydrogens for each rule can be adjusted as explained.

#### 2.4.2 Single-gen evaluation of pathway prediction

Single-gen evaluation was performed at reaction level to compare the prediction performance of models trained with automatic rules and manually curated rules. For performance evaluation, we calculated precision and recall (Equations 2 and 3). where TP and FP represent the numbers of correctly predicted and falsely predicted transformation products, respectively. It should be noted that FN only represents the transformation products documented in the dataset that cannot be predicted by models, which might be an underestimation relative to the true situation due to limitations of the analytical method used for transformation product detection. 80% of compounds were chosen to train models, while the remaining 20% were selected for testing. The evaluation was repeated 100 times for an unbiased examination of how well the model can predict new data not included during training.


(2) Precision=TPTP+FP

(3) Recall=TPTP+FN

#### 2.4.3 Multi-gen evaluation of pathway prediction

Multi-gen evaluation was proposed in a previous study (Tam *et al.* 2021) and used to evaluate prediction performance for whole pathways. In multi-gen evaluation, transformation products emerging after several generations are assigned reduced weights when calculating precision and recall because they have higher uncertainties. In addition, intermediate predicted transformation products that are not observed in the dataset are not punished as false positives if they lead to correct downstream products. Different from the previously reported version of multi-gen evaluation, in this study, unobserved products created along with correct products from the same reactions, e.g. in an oxidative cleavage or hydrolysis reaction, were not punished as false positives. This modification will result in higher precision values compared to the original version of the multi-gen algorithm. Similar to single-gen evaluation, models were trained on 80% of compounds and tested on the rest, which was repeated 100 times.

## 3 Results and discussion

### 3.1 Automatic rules

**Figure 3.**
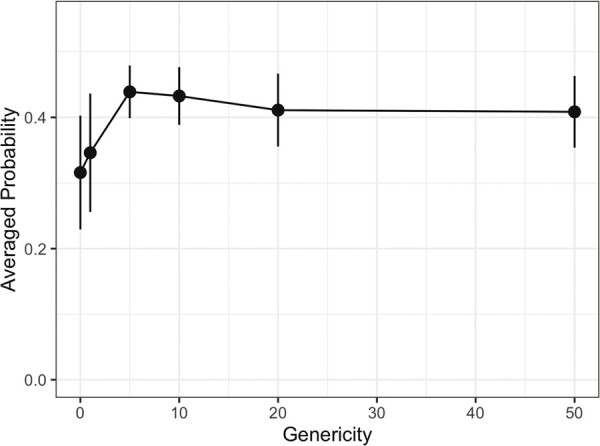
Averaged predicted probabilities of test reactions for predefined genericity levels used in automatic rule extraction. Error bars are calculated with the results from five different train and test splits (see also Supplementary Fig. S1).

Figure 3 shows the averaged probabilities of test reactions predicted by models trained with different sets of automatic rules adjusted to predefined genericity levels. High averaged probabilities indicate a high confidence of the thus trained models for the test reactions. Since all test reactions were actually experimentally observed, the genericity yielding models with highest averaged probabilities for test reactions is preferred. Among the six predefined genericities, the model trained with automatic rules generated at a genericity level of five resulted in the highest averaged probability. Hence, five was determined as the optimum genericity level for automatic rule generation and used in all the subsequent steps.

**Figure 4.**
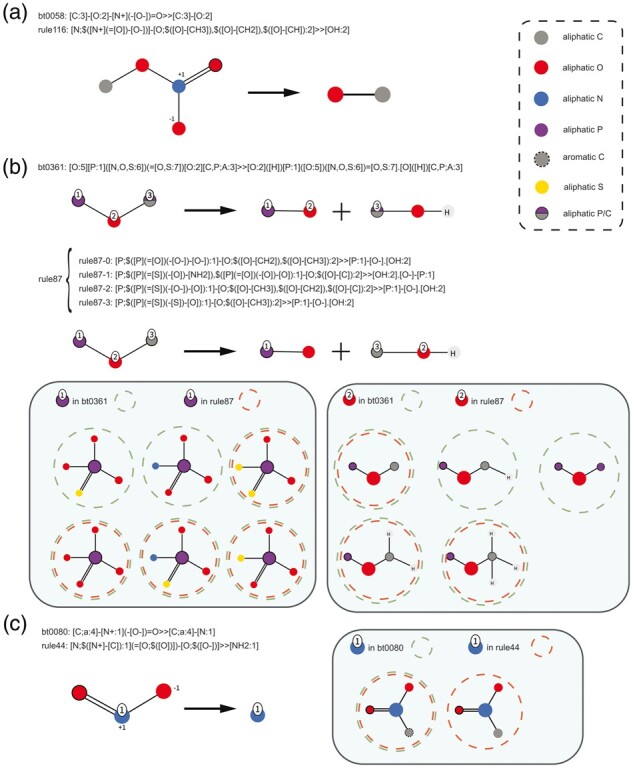
One-to-one relationships of manual btrules and automatic rules. If the substituents of atoms in the reaction centers are similar, automatic rules and manual btrules can have almost the same forms and genericities (a). However, manual btrules can have higher genericities (b) if the substituents are more diverse, or lower genericities (c) if their substituents are less diverse, while the genericities of automatic rules remain stable.

Using the optimized genericity level of 5, 143 automatic rules were extracted using the reaction data provided in the EAWAG-BBD package. They successfully covered 826 of 1132 target biotransformation reactions (73.0% coverage), while the previously 208 manually designed rules used in Eawag-BBD (Gao *et al.* 2010) and transferred into the enviPath EAWAG-BBD package, known as btrules, covered 745 reactions (65.8% coverage). The reactions covered by the two sets of rules showed a significant overlap. 641 reactions can be concurrently covered by 96 automatic rules and 116 btrules, implying that more than 86% reactions covered by btrules can also be covered by automatic rules. 86 out of 116 btrules share reactions with only one automatic rule, which is a one-to-one relationship. Manual rules with a one-to-one relationship can have almost the same form as their corresponding automatic rule, which is, e.g. the case for the hydrolysis rule for nitroesters, i.e. bt0058 and automatic rule-116 (Fig. 4). The only difference is the carbon connected to the oxygen atom in the reaction center of rule-116 cannot be quaternary, while in bt0058 it can be any carbon. However, since the genericities of manual rules are not explicitly regulated but the genericities of automatic rules are adjusted to be close to 5, btrules and corresponding automatic rules might differ in their specification of neighboring groups, even though the reaction centers are the same. As a result, btrules can have higher or lower genericity than their corresponding automatic rule in one-to-one relationships. The hydrolysis rule for phosphate/thiophosphate esters, i.e. bt0361 (genericity = 8.20), for instance, has a higher genericity than the corresponding automatic rule-87 (genericity = 5.20), while the rule for reduction of nitro groups to amino groups, i.e. bt0080 (genericity = 2.25), has lower genericity than the corresponding automatic rule-44 (genericity = 5.11) (Fig. 4). In addition to one-to-one relationships, several one-to-many relationships are observed, where the reactions covered by one manual rule are covered by several automatic rules, or the other way around. The reductive dehalogenation rule bt0029, for instance, which removes chloride, bromide, and iodine atoms from aromatic or aliphatic carbons and replaces them with a hydrogen atom, is associated with automatic rule-123, rule-137, and rule-152, which handle the dechlorination of aliphatic carbons, the dechlorination of aromatic carbons, and the debromination of aromatic carbons, respectively (Supplementary Fig. S3). None of the automatic rules covers the debromination of aliphatic carbons or deiodination in general because such reactions are not present in the EAWAG-BBD package. Conversely, reactions covered by several manual rules can also be covered by only one automatic rule. The rule for oxidation of primary alcohols to aldehydes, i.e. bt0001, for instance, and the rule for oxidation of secondary alcohols to ketone/esters, i.e. bt0002, are combined into the automatic rule-19 (Supplementary Fig. S4) because the reaction centers of these two rules are the same, both including a C–O single bond and a C–H bond.

For automatic rule updates, firstly, the 143 automatic rules extracted from the EAWAG-BBD reactions were applied to the EAWAG-SOIL reactions. 1118 of 2447 EAWAG-SOIL reactions could not be covered by any of the initial set of 143 automatic rules extracted from EAWAG-BBD and were therefore used for the rule updates. Automatic rules that had the same reaction centers as one or several uncovered reactions were automatically adapted to include the newly introduced substituents. For example, the substructure [CH](-[C])-[CH3] was added as a possible neighboring group of the carbon atom in the reaction center of automatic rule-326 to cover the reaction 0000154 in the EAWAG-SOIL package (Supplementary Fig. S5). Upon rule updating, 38 out of the 143 original automatic rules were changed and 100 new rules were created, resulting in an updated rule set including 243 automatic rules in total. With this updated set of rules, the number of not covered EAWAG-SOIL reactions dropped from 1118 to 542.

### 3.2 Evaluation of models with automatic rules extracted from EAWAG-BBD

**Figure 5.**
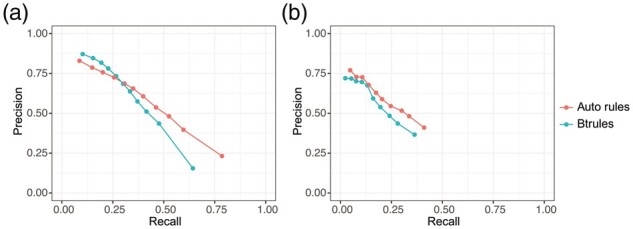
Performance of models with the EAWAG-BBD reactions in (a) single-gen evaluation and (b) multi-gen evaluation.

In Fig. 5a, results for single-gen evaluation for models generated with the original, manually extracted btrules and the newly generated automatic rules are given. Automatic rules cannot reach the same maximum precision as btrules at the highest probability threshold, but their advantages in coverage become salient at lower thresholds. The area under the curve (AUC) scores of the model trained with manual btrules and the model trained with automatic rules are 0.30 and 0.39, respectively, suggesting a better overall performance at reaction level for the model trained with the automatic rules, primarily due to the gain in recall. Similar results can be observed in models trained with more evenly split (i.e. 60%/40% and 70%/30%) train/test data (Supplementary Fig. S7).

The results of multi-gen evaluation at pathway level are visualized in Fig. 5b. AUC values for multi-gen evaluation, i.e. evaluation at pathway level, are generally lower than for single-gen evaluation because downstream reactions will never get a chance to be predicted if upstream reactions cannot be predicted, making it more challenging to obtain a high recall. Yet, similar to single-gen evaluation, the AUC score of the model trained with manual btrules (0.19) is lower than of the model trained with automatic rules (0.21), supporting the superior performance of the automatic rules over the manually curated ones at pathway level. The increase in AUC is also mainly caused by the high recall of automatic-rule-trained models.

### 3.3 Evaluation of models with automatic rules updated with EAWAG-SOIL

**Figure 6.**
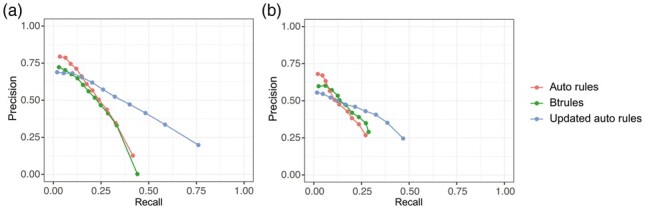
(a) Single-gen evaluation and (b) multi-gen evaluation of models against the EAWAG-SOIL reactions for three sets of rules, i.e. manually curated rules (btrules), automatic rules extracted from EAWAG-BBD data (auto rules), and automatic rules updated with EAWAG-SOIL data (updated auto rules).

Both manual btrules and automatic rules extracted from the EAWAG-BBD reactions failed to generalize well for the prediction of the EAWAG-SOIL data. According to the single-gen evaluation results shown in Fig. 6a, the AUC scores for the models trained with btrules and automatic rules and evaluated on the EAWAG-SOIL reactions were 0.19 and 0.20, respectively, which is substantially lower than the AUC scores in the single-gen evaluation of the two models trained and evaluated on the EAWAG-BBD reactions. After rule updating, an AUC score of 0.35 was achieved by the model trained with the updated automatic rules, suggesting a considerable improvement in the performance of the model with the set of updated rules.

Results for multi-gen evaluation again agreed with results for single-gen evaluation (Fig. 6b). The difference between the AUC scores for the model trained with btrules (0.13) and the model trained with automatic rules extracted from the EAWAG-BBD reactions (0.12) was tiny when evaluated against the EAWAG-SOIL data. Yet, as a consequence of the good coverage of the EAWAG-SOIL reactions by updated automatic rules, the AUC score (0.20) for the model trained with updated automatic rules improved, largely due to the increase in recall.

### 3.4 Benchmarking against other automatic rules

To enable benchmarking the performance of our automatic rules against external automatic rule sets, the single-gen evaluation was implemented with RDKit and Meka wrapper in Scikit-multilearn Python package. We compared the single-gen evaluation results on the EAWAG-BBD package for the models trained with automatic rules extracted from the EAWAG-BBD reactions and the automatic rules extracted from MetaCyc dataset (Ni *et al.* 2021). MetaCyc is a comprehensive database that contains metabolic pathways and enzymes from various domains of life, including environmental bacteria like Nitrospirae and Planctomycetes (Caspi *et al.* 2018). Since all the biotransformation reactions in the EAWAG-BBD package are decomposition reactions, i.e. one substrate reacting to one or several products, only the 255 decomposition rules were selected from the 2318 MetaCyc auto rules for the comparison. Although MetaCyc auto rules showed good generalization for KEGG and BRENDA reactions (Ni *et al.* 2021), the 255 MetaCyc decomposition rules could only cover 342 of 1132 EAWAG-BBD reactions (30.2% coverage). This most likely is the main reason also for the low AUC score of 0.11 of the prediction model trained with MetaCyc auto rules (Supplementary Fig. S6).

## 4 Conclusion

enviRule is proposed as an efficient tool for automatic extraction of reaction rules, required for rule-based biotransformation pathway prediction systems, from biotransformation reactions. In contrast to manual rules, automatic rules are extracted in a more thorough, data-driven approach and therefore no biotransformation reaction is overlooked. While this approach achieves a higher coverage of reactions compared to the previously used, manually curated rules, the genericity of rules is well controlled, and the number of rules is carefully minimized by a graph combination algorithm without over-generalization. We demonstrated that the optimum genericity of rules for the specific task of contaminant pathway prediction could be determined by comparing the predicted probabilities of test reactions from enviPath prediction models trained with rule sets that have different genericities. This optimum genericity of rules is expected to not change significantly when reactions from a similar domain are added (see, e.g. the predicted probabilities for the combined set of EAWAG-BBD and EAWAG-SOIL reactions in Supplementary Fig. S8). enviRule also allows streamlined, computationally efficient rule adaption when new reaction information is obtained, focusing only on rules that need to be updated and rules that need to be newly created, instead of running rule extraction all over again for existing and new reactions. The experimental results show that the improvement in recall for the models trained with automatic rules leads to a significant increase in the overall performance of TP prediction, compared with the models trained with manually curated rules.

Despite the remarkable achievements of enviRule, the limitations should also be noticed. Among the reaction groups clustered by the rule clusterer module of enviRule, 722 of them only have single instances. They are considered too unique for rule extraction, and hence they are skipped in the rule generator. However, some of them could contain important rules, and, upon enrichment of our reaction datasets with additional reactions, eventually more reactions might be clustered into them. For example, we are missing a rule for reductive debromination of aliphatic carbons because there are not enough examples of that type of reaction in EAWAG-BBD and EAWAG-SOIL reaction databases. One option to populate reaction clusters with additional reactions would be to augment our reaction databases with reactions from enzyme databases such as BRENDA and KEGG. However, it remains unclear which of these reactions would be observed under environmentally relevant conditions, and therefore the meaningfulness of such an approach must be carefully evaluated.

## Supplementary Material

btad407_Supplementary_Data

## Data Availability

The automatically extracted rules underlying this article, as well as 255 decomposition rules extracted from MetaCyc dataset (Ni *et al.* 2021), are available at online repositories (https://github.com/zhangky12/enviRule), and the reaction data used for rule extraction is available on https://envipath.org/.
