# Supplementary material for: enviRule: an end-to-end system for automatic extraction of reaction patterns from environmental contaminant biotransformation pathways
Source: Bioinformatics. 2023 Jun 24;39(7):btad407. doi: 10.1093/bioinformatics/btad407 (PMC10322654; doi:10.1093/bioinformatics/btad407)
Supplement: btad407_Supplementary_Data [file btad407_supplementary_data.pdf]

Supplementary Material of enviRule

Author: Kunyang Zhang[1,2, *], Kathrin Fenner[1,2]
[1]Eawag, Department of Environmental Chemistry, Überlandstrasse 133, 8600 Dübendorf, Switzerland, [2]University of Zürich, Department of Environmental Chemistry, Winterthurerstrasse 190, 8057 Zürich,Switzerland

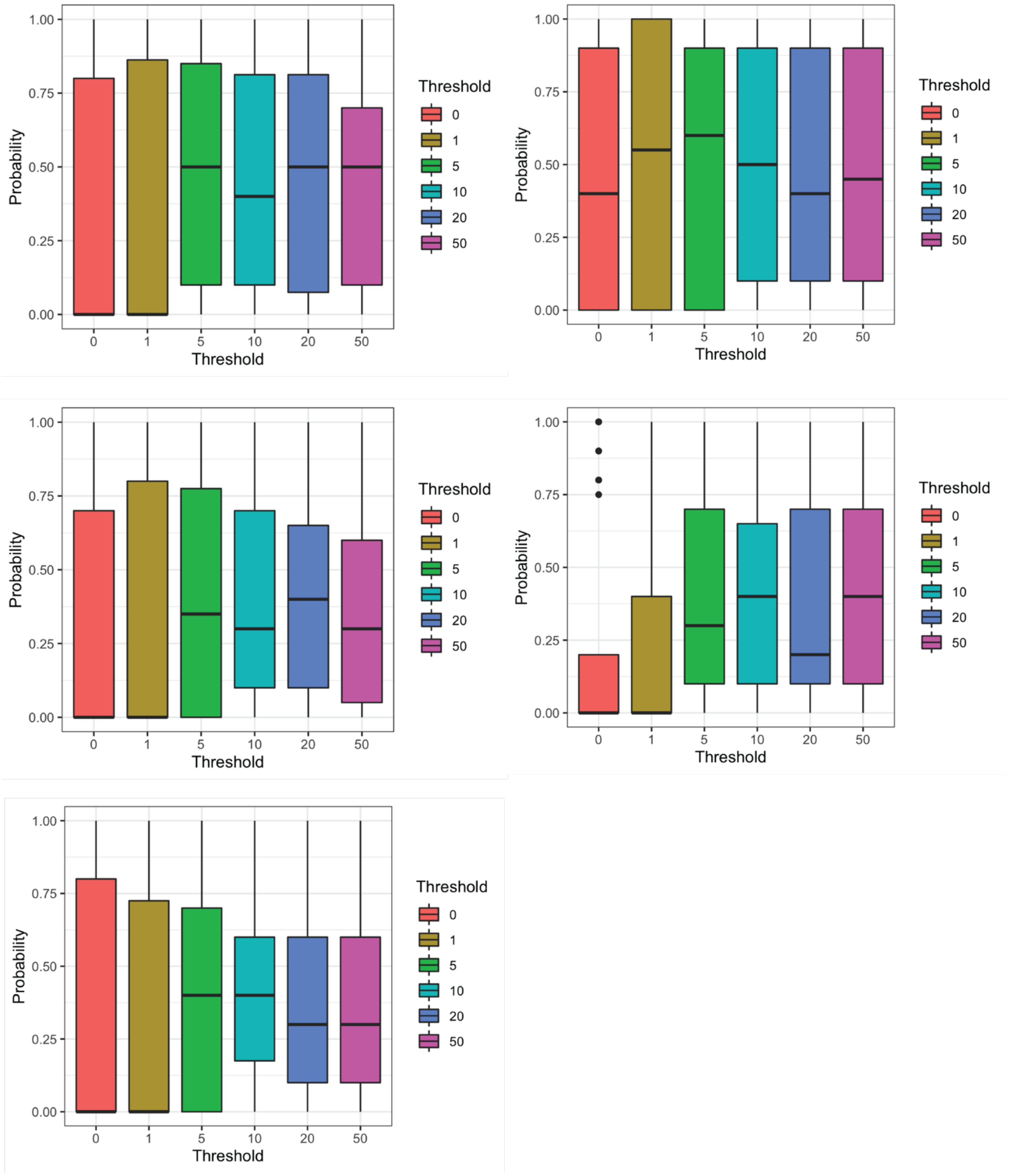

Supplementary Fig. 1. Probabilities of test reactions predicted by the models trained with different automatic rule sets. The genericity thresholds of different rule sets range from 0 to 50. Each plot shows the results of one train/test split of reactions.

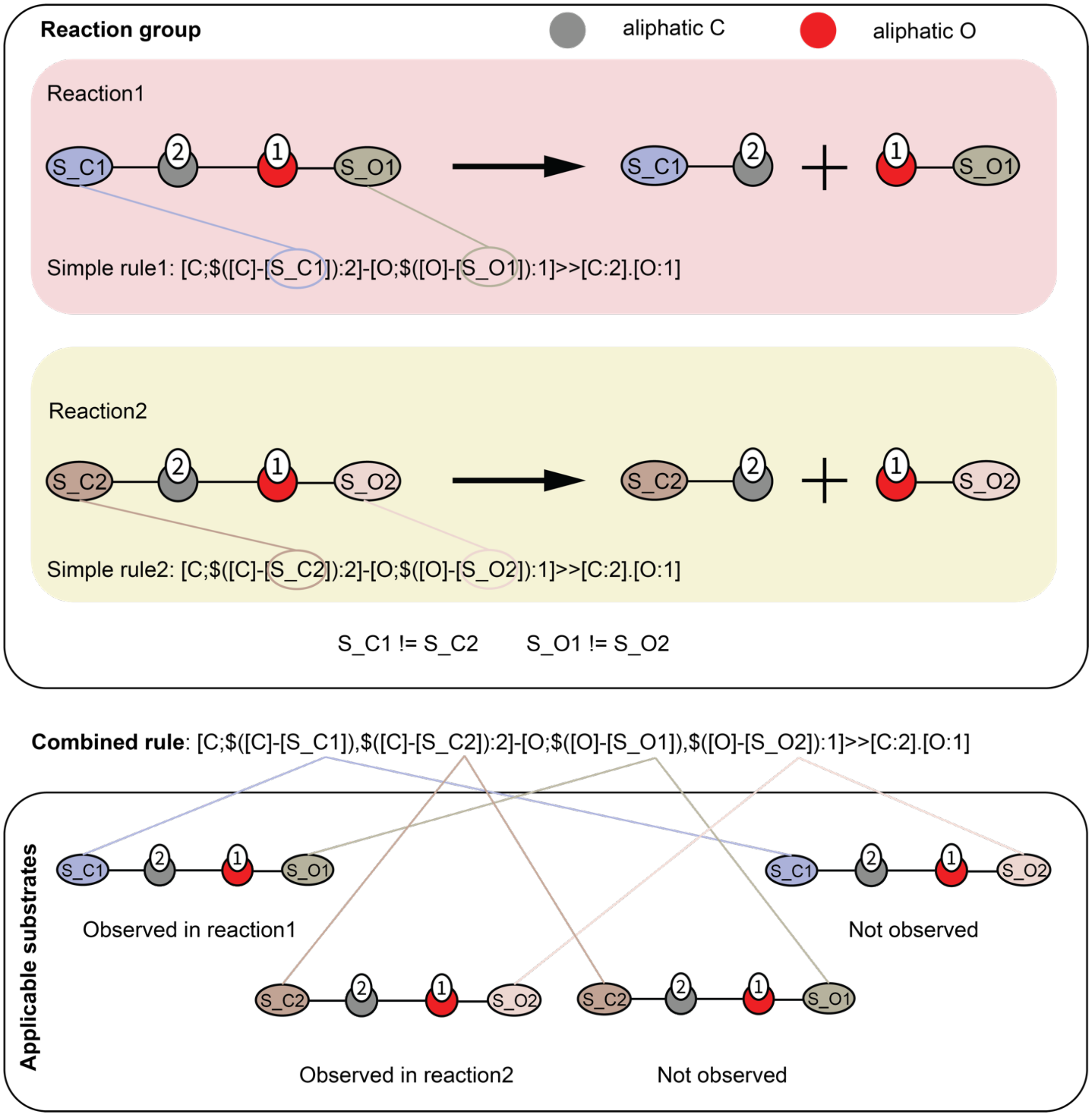

Supplementary Fig. 2. Unobserved combinations of substituents cause over-generalization of combined rules, hence combined rules can be triggered on substrates and lead to reactions that are not documented in the datasets.

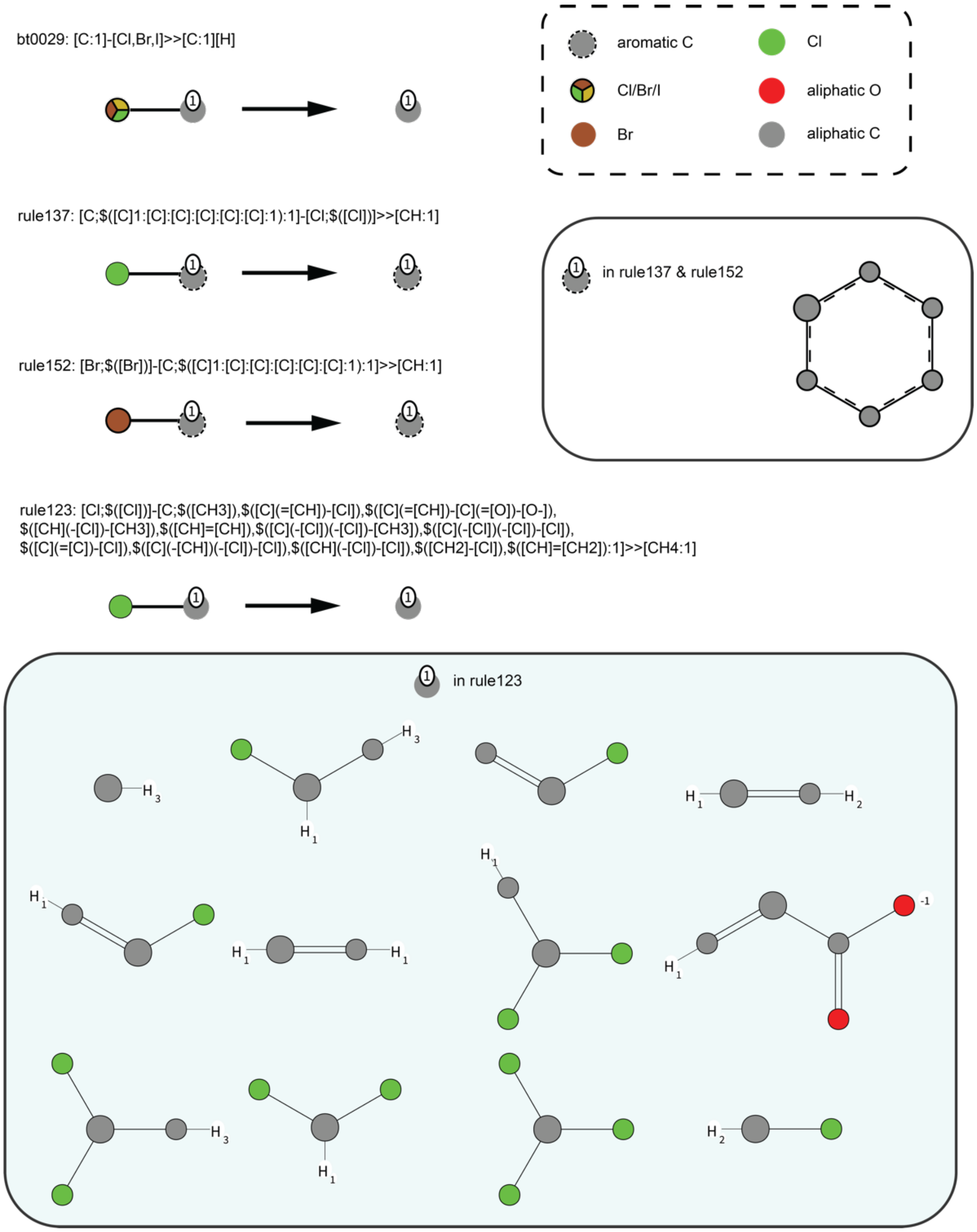

Supplementary Fig. 3. One-to-many relationships of manual btrule (bt0029) and automatic rules (rule123, rule 137, and rule 152).

bt0001: [H][#8:2][C:1]([H:5])([H])[#1,#6:6]>>[H:5][#6:1](-[#1,#6:6])=[O:2]

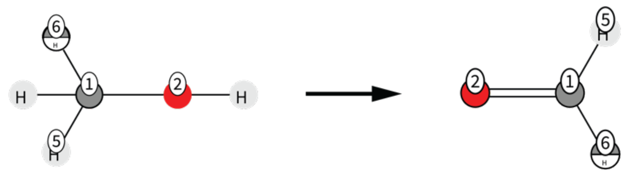

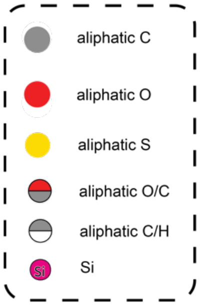

bt0002: [H][O:1][C;A;!$(CCC(O)[O-]):2]([H])([C:5])[C,O:6]>>[C:5]-[C:2](-[C,O:6])=[O:1]

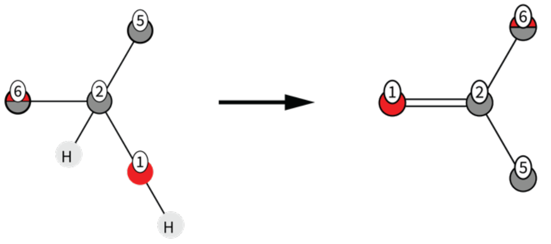

rule19: [O;$([OH]):1]([H])-[C;$([CH2]-[C]),$([CH](-[CH2])-[CH3]),$([CH](-[O])-[CH2]),$([CH](-[CH])-[C]),
$([CH](-[CH2])-[CH]),$([CH2]-[CH]),$([CH2]-[CH2]),$([CH](-[C])-[CH3]),
$([CH](-[CH])-[O]),$([CH](-[C](=[O])-[O-])-[C]),$([CH2]-[Si]),$([CH](-[C])-[C]),$([CH3]),
$([CH](-[CH2])-[CH2]),$([CH2]-[S]),$([CH2]-[CH3]):2]([H])>>[O:1]=[CH:2]

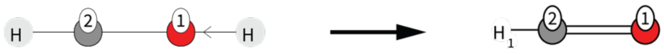

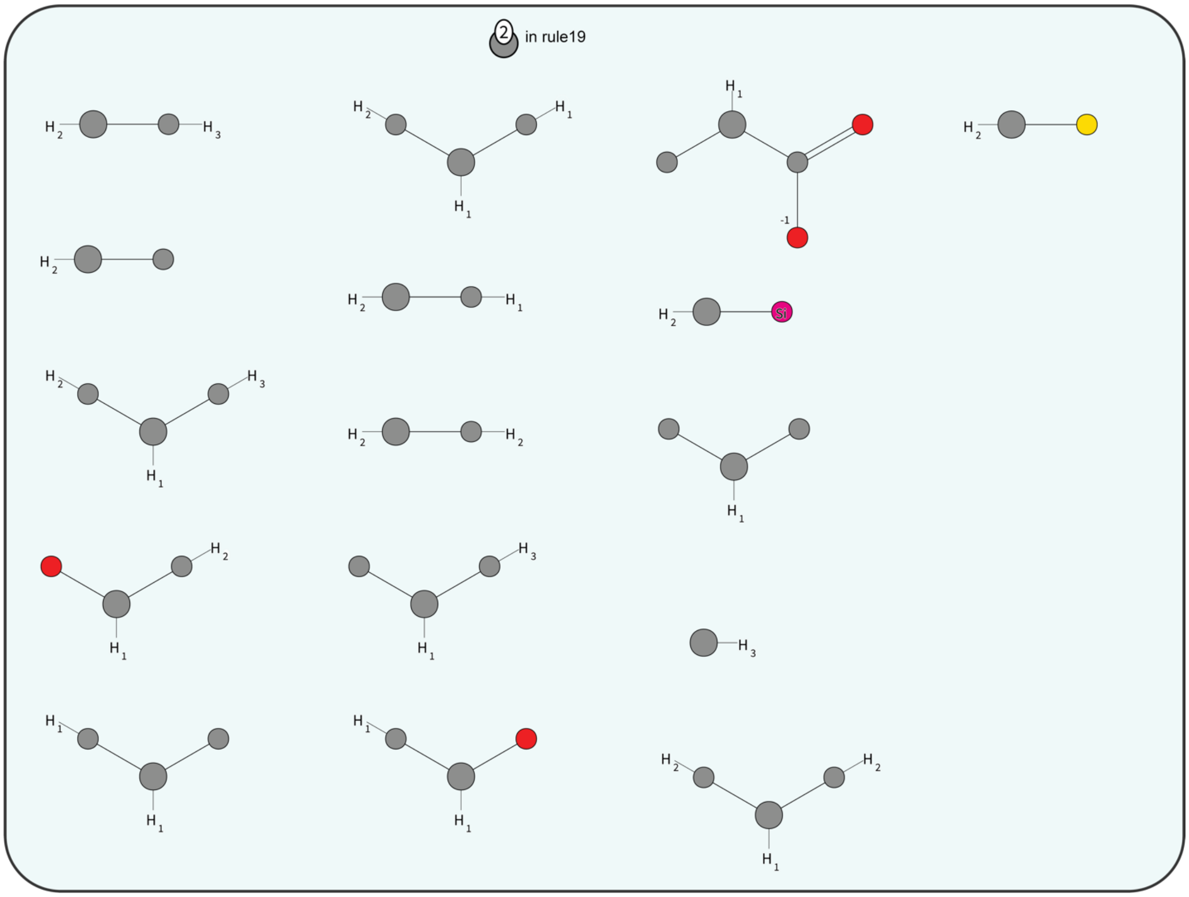

Supplementary Fig. 4. Many-to-one relationships of manual btrules (bt0001 and bt0002) and automatic rule (rule19).

rule326 (before): [C;$([CH2]-[C]),$([C](=[CH])-[CH]):1]-[N;$([NH2])]>>[CH2:1]-[OH]

rule326 (after): [C;$([CH2]-[C]),$([C](=[CH])-[CH]),**$([CH](-[C])-[CH3])**:1]-[N;$([NH2])]>>[CH2:1]-[OH]

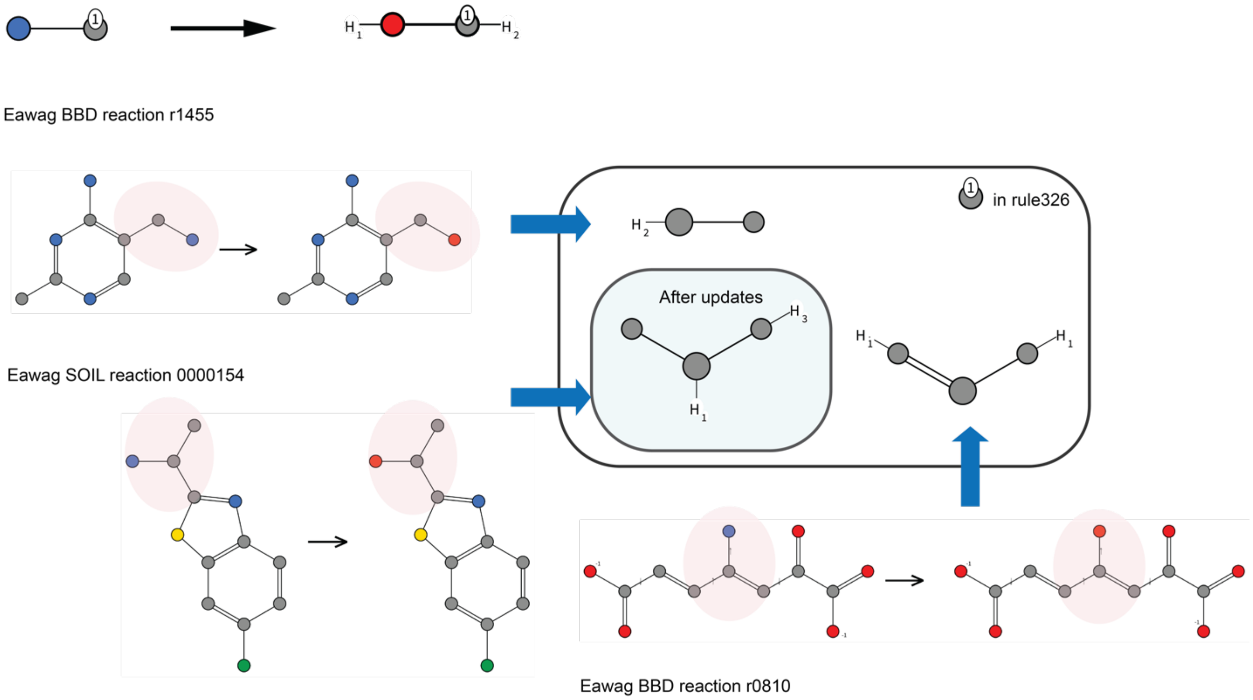

Supplementary Fig. 5. Automatic updates of rule326 with Eawag SOIL reaction 0000154.

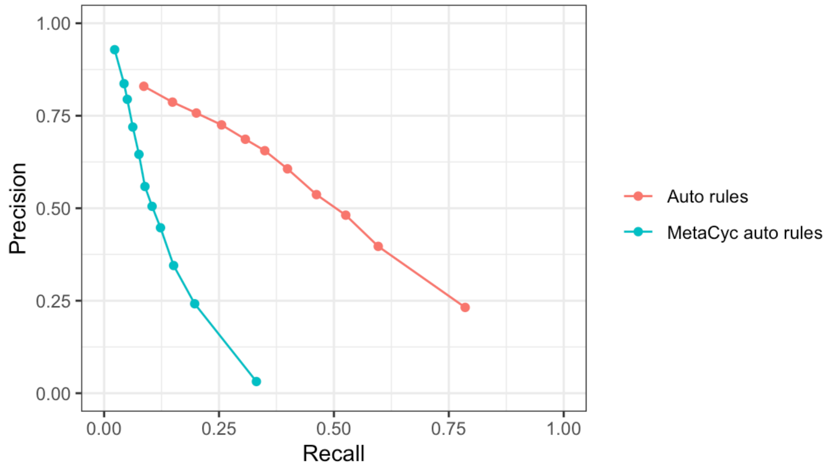

Supplementary Fig. 6. Single-gen evaluation of models trained with BBD-derived auto rules and MetaCyc auto rules against the EAWAG-BBD reactions.

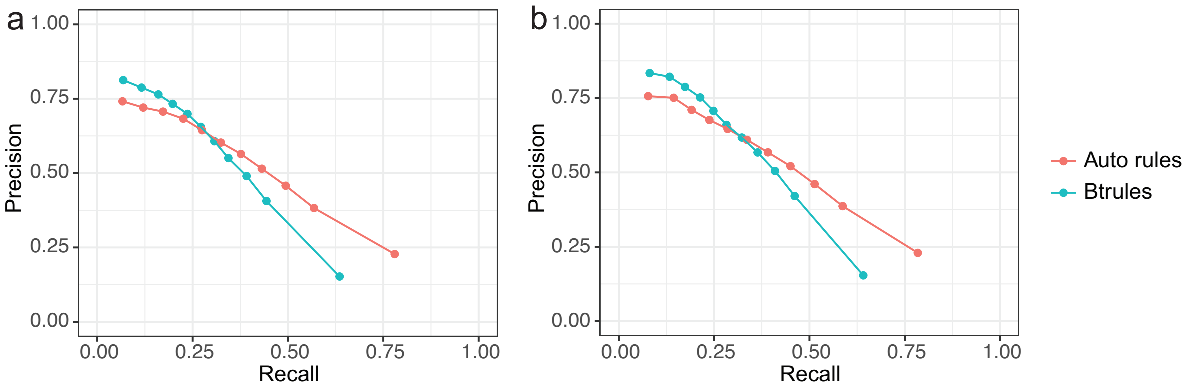

Supplementary Fig. 7. Single-gen evaluation on EAWAG-BBD reactions of models with different train/test split ratios. The AUC scores of automatic rules and manual btrules, using (a) 60% training data, are 0.36 and 0.29, respectively. While the AUC scores of automatic rules and manual btrules when using (b) 70% training data are 0.37 and 0.30.

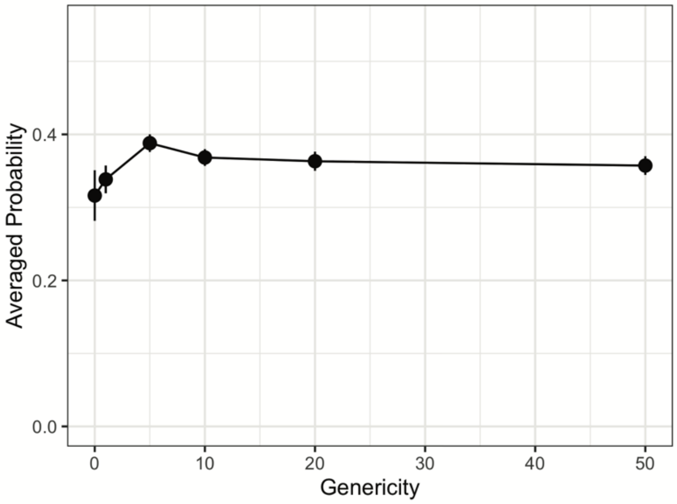

Supplementary Fig. 8. Averaged predicted probabilities of test reactions for predefined genericity levels used in automatic rule extraction from the combined set of EAWAG-BBD and EAWAG-SOIL reactions. Error bars are calculated with the results from five different train and test splits.

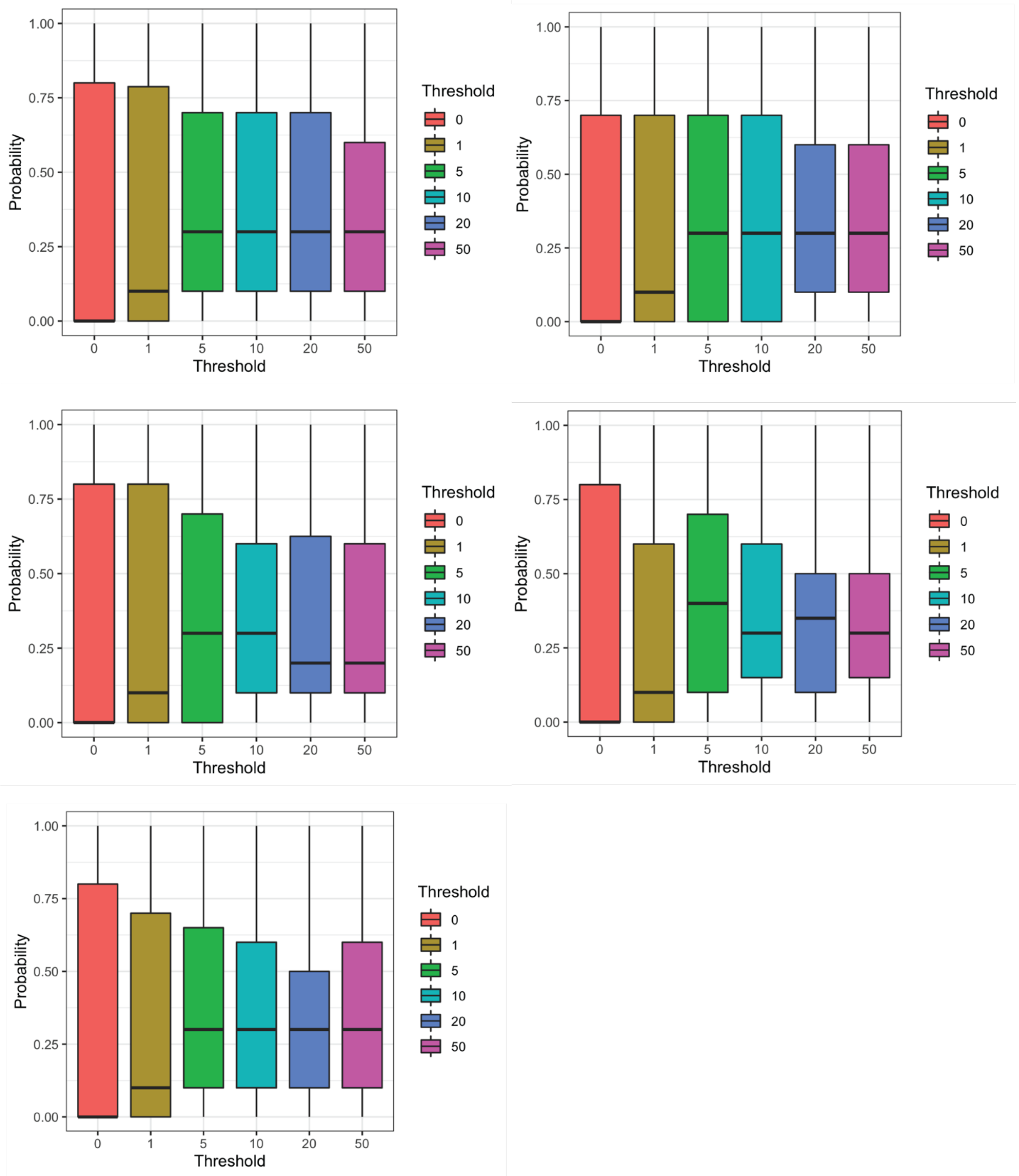

Supplementary Fig. 9. Probabilities of test reactions predicted by the models trained with different automatic rule sets extracted from combined EAWAG-BBD and EAWAG-SOIL reactions. The genericity thresholds of different rule sets range from 0 to 50. Each plot shows the results of one train/test split of reactions.
